# Impairment of Decidualization of Endometrial Stromal Cells by hsa-miR-375 Through NOX4 Targeting

**DOI:** 10.1007/s43032-022-00854-w

**Published:** 2022-01-24

**Authors:** Seong-Lan Yu, Da-Un Jeong, Yujin Kang, Tae-Hyun Kim, Sung Ki Lee, Ae-Ra Han, Jaeku Kang, Seok-Rae Park

**Affiliations:** 1 Priority Research Center, Myunggok Medical Research Institute, College of Medicine, Konyang University, Daejeon, Republic of Korea; 2 Department of Obstetrics and Gynecology, Konyang University Hospital, Daejeon, Republic of Korea; 3 I-Dream Clinic, Department of Obstetrics and Gynecology, Mizmedi Hospital, Seoul, Republic of Korea; 4 Myonggok Medical Research Center, College of Medicine, Konyang University, Daejeon, Republic of Korea; 5 Department of Pharmacology, College of Medicine, Konyang University, Daejeon, 35365 Republic of Korea; 6 Department of Microbiology, College of Medicine, Konyang University, Daejeon, 35365 Republic of Korea

**Keywords:** Endometrial stromal cells, Decidualization, miR-375, Reactive oxygen species, NOX4

## Abstract

Decidualization of the endometrial stromal cells (ESCs) is essential for successful embryo implantation. It involves the transformation of fibroblastic cells into epithelial-like cells that secrete cytokines, growth factors, and proteins necessary for implantation. Previous studies have revealed altered expression of miR-375 in the endometrium of patients with recurrent implantation failure and the ectopic stromal cells of patients with endometriosis. However, the exact molecular mechanisms, particularly the role of microRNAs (miRNAs) in the regulation of decidualization, remain elusive. In this study, we investigated whether decidualization is affected by miR-375 and its potential target(s). The findings demonstrated the downregulation of the expression of miR-375 in the secretory phase compared to its expression in the proliferative phase of the endometrium in normal donors. In contrast, it was upregulated in the secretory phase of the endometrium in infertility patients. Furthermore, during decidualization of ESCs in vitro, overexpression of miR-375 significantly reduced the transcript-level expression of forkhead box protein O1 (*FOXO1*), prolactin (*PRL*), and insulin-like growth factor binding protein-1 (*IGFBP1*), the well-known decidual cell markers. Overexpression of miR-375 also resulted in reduced decidualization-derived intracellular and mitochondrial reactive oxygen species (ROS) levels. Using the luciferase assay, we confirmed that NADPH oxidase 4 (*NOX4*) is a direct target of miR-375. Collectively, the study showed that the miR-375-mediated *NOX4* downregulation reduced ROS production and attenuated the decidualization of ESCs. It provides evidence that miR-375 is a negative regulator of decidualization and could serve as a potential target for combating infertility.

**Supplementary Information:**

The online version contains supplementary material available at 10.1007/s43032-022-00854-w.

## Introduction

Decidualization of the endometrium, the process of differentiation of human endometrial stromal cells (ESCs) during the menstrual cycle, occurs during more than 400 menstrual cycles in a woman’s lifetime. Endometrial stromal cells cause decidualization by inducing morphological and functional changes in the endometrium through increased intracellular cAMP levels after ovulation. This process is essential for successful implantation and, therefore, a successful pregnancy [1]. Decidualized human endometrial fibroblasts transform into secretory cells to produce phenotypic markers of decidual cells such as prolactin (PRL) and insulin-like growth factor binding protein-1 (IGFBP-1) [2, 3]. Reportedly, the impairment of this process is associated with a variety of pregnancy-related disorders and is a major cause of implantation failure and infertility [4].

MicroRNAs (miRNAs) are short (approximately 19–25 nucleotides in length) single-stranded non-coding RNA that regulate gene expression by post-transcriptional control of target genes. The activity of miRNAs is generally regulated by perfect or imperfect complementarity within the 3′-untranslated region (UTR) of the target mRNAs [5]. Aberrant miRNA expression has been shown to be associated with several diseases, including cancer, metabolic, and pregnancy-related disorders [6, 7]. Several studies have demonstrated the involvement of miRNAs in the regulation of decidualization. Qian et al. have reported that miR-222 is implicated in endometrial stromal cell differentiation [8], wherein Estella et al. have shown that MiR-96 and miR-135b decrease IGFBP-1 secretion by reducing forkhead box protein O1 (*FOXO1*) and homeobox A10 (*HOXA10*) expression in in vitro decidualization [9]. Furthermore, the expression of the members of the miR-200 family has been shown to be upregulated during the in vitro decidualization of ESCs, and aberrant expression of the members of the miR-200 family negatively affects decidualization [10]. MiR-375, located between the *CRYBA2* and *CCDC108* genes on human chromosome 1, plays a critical role in the biological function of the body [11]. miRNA microarray in the endometrium shows that miR-375 expression is upregulated during the window of implantation (WOI) in the endometrium of patients with recurrent implantation failure (RIF) [12]. In addition, dysregulation of miR-375 has been identified between ectopic and eutopic endometrium in endometriosis [13–16].

Nicotinamide adenine dinucleotide phosphate (NADPH) oxidase (NOX), a membrane-bound enzyme, is an important source of cellular reactive oxygen species (ROS), and the regulation of its activity is essential to maintain healthy ROS levels [17]. In the decidualization of ESCs, NOX mediates cyclic adenosine monophosphate (cAMP)-dependent decidualization [18]. Reportedly, NOX4 is highly expressed in the secretory phase compared to that in the proliferative phase of the endometrium [19], and its silencing inhibits the expression of PRL and IGFBP1 [18]. Although the integral role of NOX4 activation and ROS signaling in initiating the endometrial decidual transformation of human ESCs (HESCs) has been reported [18], the regulatory mechanisms of miR-375 related to endometrial decidualization have not been explored.

In this study, we aimed to investigate the correlation between miR-375 expression and decidualization in ESCs and inspect the importance of the miR-375-*NOX4* axis in decidualization. The study demonstrates that the NOX4 involved in ROS production could be a direct target of miR-375.

## Materials and Methods

### Collection of Human Endometrial Tissue

Endometrial samples were collected during the proliferative or secretory phase of the menstrual cycle from participants at the Konyang university hospital. Samples representing infertility were collected from participants at the MizMedi hospital. The menstrual stage of the endometrial samples and samples with pathology suggesting endometrial diseases were confirmed by an experienced gynecological pathologist. The volunteer had self-reported regular, normal (21–35 days) menstrual cycles. The proliferative phase samples were collected at 9–11 menstrual cycle days (mcd), and the secretory phase samples were collected at 20–24 mcd in the mid-secretory phase. Moreover, the infertility samples were collected at 20–22 mcd in the mid-secretory phase from patients of normal menstrual cycles (21–42 days) (Table 1). None of the infertility patients were undergoing hormone therapy during sample collection.

**Table 1:** Characteristics of endometrium donors for quantitative reverse transcription polymerase chain reaction (qRT-PCR)

Variables/group	Proliferative phase (9 ~ 11 mcd; *n* = 9)	Secretory phase (20–24 mcd; *n* = 8)	Infertility (20–22 mcd; *n* = 7)	*p* value
Age (yrs)	37.0 ± 3.0	37.4 ± 2.5	38.7 ± 3.5	0.60
BMI (kg/m^2^)	22.8 ± 4.4	22.9 ± 2.9	22.9 ± 4.0	0.98
No. of live birth (n)	2.1 ± 0.6	1.9 ± 0.4	0.1 ± 0.4	0.0004
No. of abortion (n)	0.1 ± 0.3	0.4 ± 0.5	0.9 ± 1.2	0.27
Average menstrual cycle (days)	30 ± 1.3	29.8 ± 1.2	32.7 ± 7.2	0.4561

*p* values were determined using the one-way ANOVA test. Data are presented as the mean ± SD

This study was approved by the Bioethics Committee of Konyang University Hospital (IRB file No. KYUH 2018–11-007) and MizMedi hospital (IRB file No. MMIRB 2018–3). The participants signed informed consent.

### Cell Culture, In Vitro Decidualization, and Cell Transfection

Immortalized non-neoplastic human endometrial stromal cells (T HESCs) were cultured in the DMEM/F-12 medium without phenol red (Sigma, USA), supplemented with 10% fetal bovine serum (FBS; GIBCO, USA), 1% insulin transferrin selenium (ITS) + Premix (Corning 3454352, USA), and 500 ng/mL puromycin (InvivoGen, USA). Cells were maintained at 37 ℃ in a humidified atmosphere with 5% CO_2_.

For in vitro decidualization, T HESCs were maintained for 6 days in the DMEM/F-12 medium containing 1 μM medroxyprogesterone 17-acetate (MPA; Sigma) and 0.5 mM 8-bromo-cyclic adenosine monophosphate (8-bromo-cAMP; Sigma), 2% FBS, and 1% ITS + Premix. The culture medium was replaced with a fresh medium every 2 days.

NOX4 siRNA, the miR-375 mimic, and the miR-375 inhibitor were transfected using Lipofectamine RNAiMAX (Thermo Fisher Scientific, USA) into T HESCs, following the manufacturer’s recommended protocol.

### RNA Isolation and Quantitative Reverse Transcription Polymerase Chain Reaction (qRT-PCR) Analysis

Total RNA was isolated from T HESCs or endometrial tissue biopsies using TRIzol® reagent (Ambion, Thermo Fisher Scientific) according to the manufacturer’s instructions. To investigate the mRNA expression, cDNA was synthesized from total RNA using Moloney Murine Leukemia Virus (M-MLV) reverse transcriptase (Promega, USA). Quantitative real-time PCR was performed using iQ SYBR Green Supermix (BioRad) for the *IGFBP1, PRL*, *FOXO1*, *NOX4*, and *GAPDH* genes using a CFX 96 qPCR (BioRad Laboratories, USA). The primers used for real-time PCR are listed in Supplementary Table 1. The following PCR amplification conditions were used: an initial denaturation step at 95 ℃ for 3 m followed by 40 cycles of denaturation at 95 ℃ for 10 s, annealing for 10 s using suitable primer sets, and extension at 72 ℃ for 10 s.

To determine the relative miR-375 expression, cDNA was synthesized using the TaqMan miRNA Reverse transcription kit (Thermo Fisher Scientific, USA), with a reverse transcription miR-375 or RNU6B primer (Thermo Fisher Scientific, USA), according to the manufacturer’s instructions. Then quantitative real-time PCR was performed using TaqMan Master Mix II and TaqMan miRNA assay primer (Thermo Fisher Scientific, USA). The 2^−ΔΔct^ method was used to calculate mRNA or miRNA expression levels using the reference gene.

### Western Blotting

The T HESCs were lysed using lysis buffer (Jubiotech, Korea) containing protease and phosphatase inhibitor (Roche, Swiss). The protein concentrations were measured using the bicinchoninic acid (BCA) assay (Thermo Fisher Scientific). Proteins in cell lysate were resolved by 10% sodium dodecyl sulfate–polyacrylamide gel electrophoresis (SDS-PAGE) and transferred to polyvinylidene difluoride (PVDF) membranes (Millipore, Billerica, MA, USA). The blots were then blocked with 5% skim milk (Difco, USA) and probed overnight with primary antibodies at 4 ℃. The primary antibodies used in this study included FOXO1 (1:1000, cell signaling, #2880), NOX4 (1:1000, Abcam, ab109225), and GAPDH (1:5000, cell signaling, #5174). After probing with primary antibodies, the membrane was incubated with horseradish peroxidase-conjugated anti-rabbit secondary antibodies (1:5000, Millipore, AP132P). The expression of the target proteins was determined using an enhanced chemiluminescence kit (Thermo Fisher Scientific).

### Enzyme-Linked Immunosorbent assay (ELISA)

The conditioned media were collected after stimulation for decidualization of T HESCs. The IGFBP1 and PRL levels were measured using the manufacturer’s instructions provided along with the ELISA kit (R&D Systems, USA). The optical density was read using a BioTek microplate reader (BioTek instrument, USA).

### Plasmid Construction and Luciferase Reporter Assay

To investigate whether miR-375 modulates *NOX4* expression by binding to its 3´UTR, the *NOX4* 3´UTR was amplified using a forward primer containing an *XhoI* restriction site (5´-CCA CTC GAG TGC CAT GAA GCA GGA CTC TA-3´), and a reverse primer containing a *NotI* restriction site (5´-CCA GCG GCC GCC AGA GTC TTG TGC TGT GTT TTC A-3´). The *NOX4* 3´UTR region was cloned into the dual-luciferase psiCHECK2 vector (Promega, Madison, WI). Mutagenesis of the binding site of miR-375 was accomplished using the KOD Plus Mutagenesis kit (Toyobo, Osaka, Japan). The sequence of the cloned vectors was verified using DNA sequencing. The cloned vector and the miR-375 mimic were co-transfected into T HESCs using the Lipofectamine 3000 (Thermo Fisher Scientific). Then, the transfected T HESCs were cultured. Afterward, luciferase activity in the transfected cells was measured using the dual-luciferase reporter assay system following the Promega instruction manual (Promega, USA). Luminescent activities of firefly luciferase were used as the internal control for the normalization of transfection efficiency. Luciferase assays were performed in triplicate.

### Measurement of ROS

To measure the intracellular ROS levels, T HESCs were seeded into the plate and incubated after stimulation with 0.5 mM 8-bromo-cAMP and 1 μM MPA for decidualization. After harvesting the cells, the cells were treated with 25 μM 2´,7´-dichlorodihydrofluorescein diacetate (DCF-DA) for 15 min in the dark at 37 ℃. We estimated the oxidation of the dye by measuring 10,000 events per sample using flow cytometry (FACS, Beckman).

To measure the mitochondrial ROS levels, T HESCs were cultured for decidualization, with 0.5 mM 8-bromo-cAMP and 1 μM MPA, and incubated with 5 μM Mitosox™ Red (Thermo Fisher Scientific) for 10 min in the dark at 37 ℃. The cells were visualized using confocal microscopy (Carl Zeiss, Germany), and the fluorescence intensity was determined using ImageJ (http://imagej.nih.gov.ij).

### Statistical Analyses

The data are presented as the mean ± standard error of the mean (SEM). All experiments were performed in triplicate. The results were analyzed using Student’s *t* test or Mann–Whitney test. Results were considered statistically significant at *P* < 0.05 or *P* < 0.01.

## Results

### Inverse Relationship of miR-375 with Decidualization

**Fig. 1:**
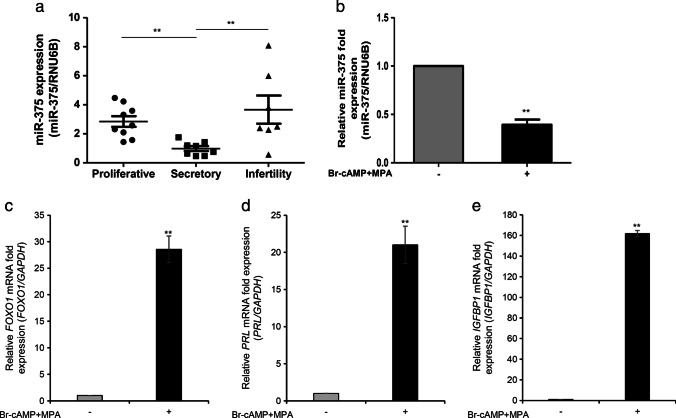
Downregulation of miR-375 expression during decidualization of endometrial stromal cells; **a** Expression patterns of miR-375 in samples collected from the proliferative, secretory phase of normal endometrial biopsies, and secretory phase of endometrial biopsies of patients with infertility; **b** miR-375 expression in decidualized T HESCs stimulated with 8-br-cAMP and MPA. mRNA expression of decidual markers **c** *FOXO1*, **d** *PRL*, and **e** *IGFBP-1* in decidualized T HESCs stimulated with 8-br-cAMP and MPA. All data represent mean ± SEM from three independent experiments. **P < 0.01

The microarray-based miRNA profiling studies have shown that miR-375 is upregulated during the WOI in the endometrium of RIF patients [12]. Therefore, we investigated the change in miR-375 expression between the proliferative and secretory phases of the endometrium to find a link to decidualization. In the normal endometrial tissue biopsy samples, the quantitative RT-PCR revealed downregulation of the expression of miR-375 in the secretory phase samples compared with that in the proliferative phase samples. On the contrary, its expression was upregulated in the secretory phase samples collected from the endometrial biopsies of the patients with infertility (Fig. 1a). Further decidualization of T HESCs in vitro by treating T HESCs with cAMP and MPA demonstrated significant downregulation of the expression of miR-375 in the decidualized T HESCs (Fig. 1b). In addition, the significant upregulation of decidual markers, including *FOXO1*, *PRL*, and *IGFBP1*, confirmed the decidualization status of T HESCs (Fig. 1c–e). These results revealing an inverse relationship between miR-375 expression and decidualization indicate that the suppression of expression of miR-375 is essential for decidualization.

### Inhibition of T HESCs Decidualization Through miR-375 Overexpression

**Fig. 2:**
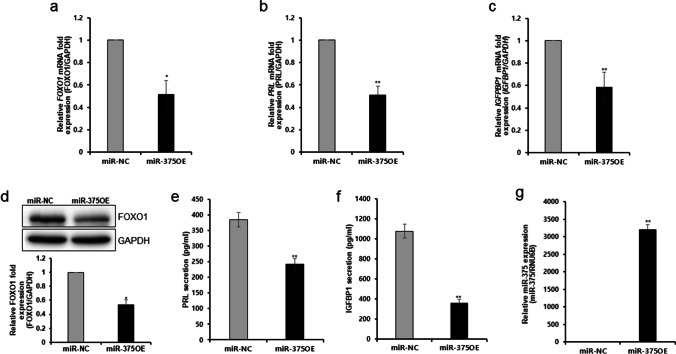
Inhibition of in vitro decidualization of endometrial stromal cells by miR-375 overexpression. **a**–**c** Effect of miR-375 overexpression on decidual markers on mRNA expression of **a** *FOXO1*, **b** *PRL*, and **c** *IGFBP-1* in decidualized T HESCs stimulated with 8-br-cAMP and MPA; **d** FOXO1 protein expression in T HESCs with miR-375-overexpression. Effects of miR-375-overexpression on secretion **e** PRL and **f** IGFBP-1 T HESCs; **g** miR-375 expression in T HESCs transfected with miR-375 mimic. All data represent mean ± SEM from three independent experiments. *P < 0.05, **P < 0.01

To confirm the role of mir-375 on decidualization, the effect of miR-375 overexpression on the mRNA and protein expression of the decidual markers, FOXO1*,* PRL, and IGFBP-1 was investigated. The findings demonstrated that overexpression of miR-375 significantly reduced the transcript-level expression of *FOXO1*, *PRL*, and *IGFBP1* in decidual cells than that of the negative control (Fig. 2a–c). miR-375 overexpression also inhibited the expression of FOXO1 protein significantly (Fig. 2d). Further, estimation of the effects of mir-375 overexpression on the secretion of PRL and IGFBP1 in the decidualized cells using ELISA revealed a significant reduction of the expression these two marker proteins in T HESCs transfected with miR-375 mimic (Fig. 2e, f). These results indicate that miR-375 overexpression suppresses the decidualization of ESCs.

### Inhibition of ROS Production by miR-375 During In Vitro Decidualization in T HESCs

**Fig. 3:**
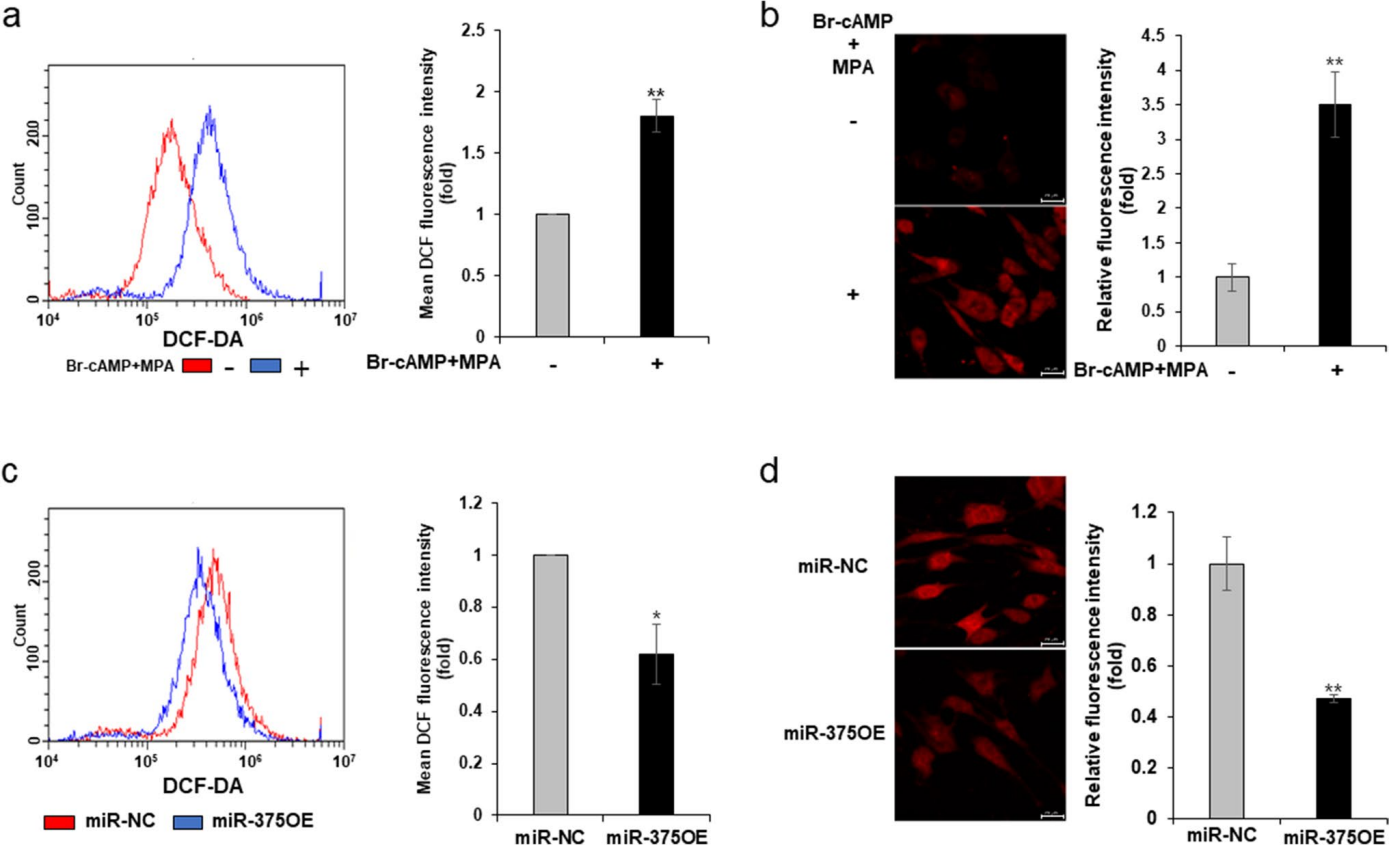
Inhibition of ROS levels by miR-375 overexpression during in vitro decidualization of T HESCs. **a** Intracellular ROS levels in decidualized T HESCs induced with 8-br-cAMP and MPA; **b** mitochondrial ROS levels in decidualized T HESCs induced with 8-br-cAMP and MPA; **c** effect of miR-375 overexpression on intracellular ROS levels in decidualized T HESCs stimulated with 8-br-cAMP and MPA; **d** effect of miR-375 overexpression on mitochondrial ROS levels in decidualized T HESCs stimulated with 8-br-cAMP and MPA. All data represent mean ± SEM from three independent experiments. *P < 0.05, **P < 0.01

As the resistance to oxidative stress and the levels of intracellular ROS remarkably increase during decidualization of ESCs [20, 21], we analyzed the intracellular and mitochondrial ROS levels in decidualized T HESCs in vitro. DCFDA oxidation levels were significantly higher in decidualized T HESCs stimulated with 8-br-cAMP and MPA (decidualized) than in those grown in the absence of 8-br-cAMP and MPA (control) (Fig. 3a). The level of mitochondrial ROS was also higher in the decidualized condition than in the control condition (Fig. 3b).

Furthermore, the overexpression of miR-375 in T HESCs stimulated with 8-br-cAMP and MPA exhibited reduced intracellular (*P* < 0.05) and mitochondrial (*P* < 0.01) ROS production (Fig. 3c, d) than in those grown without 8-br-cAMP and MPA. These results suggest that the target gene of miR-375 could be associated with ROS production.

### NOX4: a Direct Target of miR-375

**Fig. 4:**
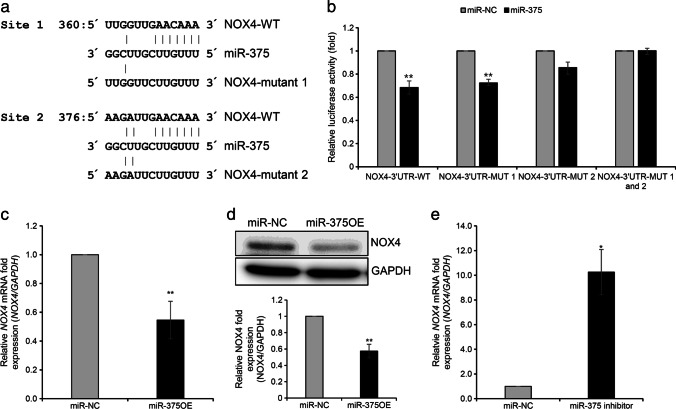
NOX4: direct target gene of miR-375. **a** Schematic representation of predicted target sequence of miR-375 and mutant sequence in the 3´UTR of *NOX4* gene; **b** relative luciferase activity of NOX4-3´UTR and mutant reporter through miR-375 overexpression; **c** expression changes of *NOX4* mRNA through miR-375 overexpression in T HESCs; **d** expression changes of NOX4 protein through miR-375 overexpression in T HESCs; **e** expression changes of *NOX4* mRNA through miR-375 inhibition in T HESCs. All data represent mean ± SEM from three independent experiments. *P < 0.05, **P < 0.01

Next, to identify a direct target of miR-375 associated with ROS production, we searched the miRDB database, an online database for miRNA target prediction and functional annotations (http://www.mirdb.org). The search identified 269 genes targeted by miR-375, including the *NOX4* gene. Reportedly, *NOX4* plays an important role in regulating the levels of intracellular ROS and changes in the expression of decidual markers PRL and IGFBP-1 in HESCs [18]. Therefore, we selected *NOX4* for further analyses. Two binding sites were predicted for miR-375 between nt 2226 and 2254 in the 3′UTR of *NOX4* mRNA (NM_016931; Fig. 4a). To confirm the direct interaction between miR-375 and *NOX4*, we performed a dual-luciferase reporter assay with the 3´UTR of *NOX4* in T HESCs. The overexpression of miR-375 significantly inhibited the luciferase activity of the mutant at the first binding site, whereas the reduction in luciferase activity of the mutant at the second binding site was non-significant. Moreover, when the reporter at the two binding sites of miR-375 was mutated, no change in luciferase activity was observed. Therefore, we suggest that miR-375 binds onto the second binding site of *NOX4* 3´UTR (Fig. 4b). Further, the qRT-PCR and western blotting analyses demonstrated that NOX4 mRNA and protein expression were significantly reduced by miR-375 overexpression (Fig. 4c, d). In contrast, inhibition of miR-375 significantly increased the mRNA expression of *NOX4* in T HESCs (Fig. 4e). These results indicate the crucial role of mir-375 in regulating *NOX4* expression.

### Significance of NOX4 for T HESCs Decidualization

**Fig. 5:**
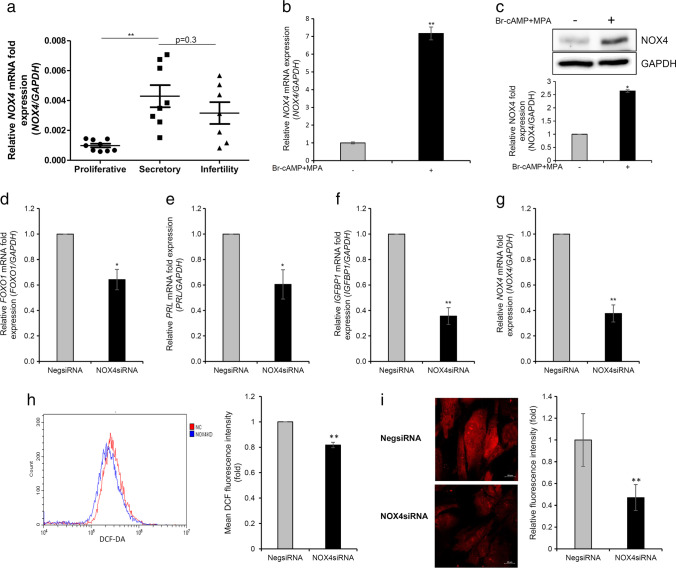
Inhibition of in vitro decidualization of endometrial stromal cells through *NOX4* knockdown. **a** Expression patterns of *NOX4* mRNA in the proliferative and secretory phase of normal endometrial tissues and secretory phase of endometrial tissues collected from patients with infertility; **b** expression changes in *NOX4* mRNA in decidualized T HESCs stimulated with 8-br-cAMP and MPA; **c** expression changes of NOX4 protein in decidualized T HESCs stimulated with 8-br-cAMP and MPA. Effect of *NOX4* knockdown on decidual markers **d** *FOXO1*, **e** *PRL*, and **f** *IGFBP-1* mRNA expression in decidualized T HESCs stimulated with 8-br-cAMP and MPA; **g** *NOX4* mRNA expression in T HESCs transfected with NOX4 siRNA; **h** effect of *NOX4* knockdown on intracellular ROS levels in decidualized T HESCs stimulated with 8-br-cAMP and MPA; **i** Effect of *NOX4* knockdown on mitochondrial ROS level in decidualized T HESCs stimulated with 8-br-cAMP and MPA. All data represent mean ± SEM from three independent experiments. *P < 0.05, **P < 0.01

We have shown that in normal endometrial biopsies, the expression of miR-375 was decreased in the secretory phase than in the proliferative phase of the endometrium (Fig. 1a). Therefore, to unravel the relationship between the expression of *NOX4* and decidualization, we analyzed the difference in the expression of *NOX4* between the proliferative and secretory phases of the endometrium. *NOX4* expression was significantly increased in the secretory phase compared to that in the proliferative phase of the endometrium. Moreover, *NOX4* expression was downregulated in the secretory phase of endometrium in the samples collected from patients with infertility (although not significantly compared to normal secretory phase) (Fig. 5a). The expression of *NOX4* in the proliferative phase of the endometrium showed an inverse pattern with respect to miR-375 expression (Figs. 1a and 5c). The mRNA and protein expression of NOX4 were significantly increased in T HESCs after in vitro cAMP- and MPA-induced decidualization (Fig. 5b, c). Furthermore, *NOX4* knockdown in T HESCs attenuated the expression of decidual markers *FOXO1*, *PRL*, and *IGFBP-1* (Fig. 5d–f). *NOX4* knockdown also reduced intracellular and mitochondrial ROS levels during in vitro decidualization (Fig. 5h, i). Collectively, these results suggested that miR-375-mediated expression changes in *NOX4* could be related to the decidualization of ESCs.

## Discussion

The uterine ESCs transform into decidual cells during decidualization, which is critical for establishing and maintaining pregnancy, as aberrant decidualization leads to pregnancy loss [22]. Several studies have reported the differential expression of several miRNAs before and after human endometrial stromal decidualization [8, 9, 23]. In addition, it has also been reported that normal ESCs and endometriotic cyst stromal cells are regulated by different mechanisms of miRNA [24], suggesting that miRNAs play important roles in human endometrial stromal decidualization. To the best of our knowledge, we are the first to provide evidence for the crucial role of miR-375 in endometrial decidualization. We demonstrate that the upregulation of miR-375 in HESCs suppresses the decidualization of these cells in vitro and confirm that *NOX4* is a direct target of miR-375.

Previous studies have reported that miR-375 plays an important role in the formation of pancreatic beta-cells [25]. The progesterone receptor (PGR) has been identified as a direct target of miR-375 in the endometrium of rhesus monkeys [26]. Rekker et al. reported aberrant miR-375 expression in ectopic stromal cells of an endometriosis patient [16]. Similarly, other miRNAs have also been reported to be involved in decidualization. miR-181a regulates the decidualization of ESCs by targeting the Kruppel-like factor 12 (KLF12) [27], while miR-542-mediated IGFBP-1 downregulation is associated with decidualization [28]. miR-194-3p has also been reported to have a relationship with decidualization via the downregulation of PGR [29]. Recently, Qu et al. have reported that miR-542-3p inhibits decidualization through the downregulation of the ILK pathway [30]. In our study, miR-375 was found to be a negative regulator of decidualization in HESCs.

During the transformation of ESCs from their normal state into a decidualized state, the decidual stromal cells generate ROS and resist cellular stress signals, to maintain the feto-maternal interface [31]. Cu, Zn-superoxide dismutase (SODs), and mitochondrial Mn-SOD are specific enzymes that scavenge superoxide radicals, the major form of ROS, and are generated after differentiation of the ESCs [32, 33]. Decidualization of ESCs also increases resistance to hydrogen peroxide-induced oxidative stress [20]. Decidual stromal cells differentiated from ESCs transform into protein-secreting cells and induce endoplasmic reticulum (ER) stress by accumulating misfolded proteins to activate secretory pathways in the ER. Consequently, ER stress induces ROS, which is involved in decidualization [34]. In addition, ESCs subjected to decidualization stimuli, such as 8-br-cAMP, MPA, and PGE2, show markedly elevated ROS levels [21]. Recently, resveratrol, well known as an antioxidant and anti-inflammatory agent, has been shown to inhibit decidualization through the downregulation of decidual markers [35]. In addition, oxidative stress increased FOXO1 expression via JNK in follicular granulosa cells in mice [36, 37]. FOXO1 has been reported to be an important factor in the decidualization of HESCs, and FOXO1 knockdown attenuates IGFBP-1 and PRL expression, the well-known decidual markers [38]. The NOX family enzymes are considered the major ROS producers in cells. Inhibition of NOX attenuates cAMP-dependent decidualization, and the silencing of NOX4 (among the NOX family members) inhibits the expression of IGFBP1 and PRL [18]. Degasper et al. have also suggested that NOX4 may play an important role in secretory transformation because it exhibits significantly higher expression in the secretory phase than in the proliferative phase of the endometrium [19]. In this study, we demonstrated that miR-375 downregulates NOX4 expression, and the downregulated NOX4 attenuates ROS production in ESCs. Taken together, we inferred that upregulation of miR-375 and downregulation of NOX4 in the endometrium inhibit decidualization leading to infertility and thereby suggest that miR-375/NOX4 axis plays an important role in pregnancy and could be a potential target for combating infertility. However, further investigations are required to ascertain the physiological and clinical relevance of the miR-375/NOX4 axis in infertility.

There are some limitations to this study. The sample of the same menstrual stage was small in size. The sample needs more refined timing across the menstrual cycle. However, more time is needed for refined timing sample collection, and it is very difficult to obtain sufficient endometrium samples for research owing to ethical restrictions. We are currently collecting endometrium samples to unravel the pathways and interactions of the miR-375/NOX4 axis with other critical mechanisms in decidualization to determine their precise relevance to combat infertility.

## Conclusion

This study, for the first time, offers evidence that miR-375 binds directly to the 3′UTR of NOX4 in ESCs, and the miR-375-mediated NOX4 downregulation reduces ROS production, which consequently attenuates the decidualization of ESCs. These results confirmed that miR-375 acts as a negative regulator of decidualization and could be a potential target for combating infertility.

## Supplementary Information

Below is the link to the electronic supplementary material.

Supplementary file1 (DOCX 15 KB)

## Data Availability

All data generated or analyzed during this study are included in this published article and its supplementary information files.
