# Supplementary material for: Impairment of Decidualization of Endometrial Stromal Cells by hsa-miR-375 Through NOX4 Targeting
Source: Reprod Sci. 2022 Jan 24;29(11):3212–21. doi: 10.1007/s43032-022-00854-w (PMC9646565; doi:10.1007/s43032-022-00854-w)
Supplement: Supplementary file 1 — Supplementary file1 (DOCX 15 KB) [file 43032_2022_854_MOESM1_ESM.docx]

Supplementary table 1. Primer sequences for qRT-PCR (quantitative reverse transcription-polymer chain reaction)

| Gene | Primer sequences | Annealing temperature | | Size of amplicon (bp) |
| --- | --- | --- | --- | --- |
| IGFBP1 | Forward: 5´- cgaaggctctccatgtcacca -3´  Reverse: 5´- tgtctcctgtgccttggctaaac-3´ | | 60°C | 98 |
| PRL | Forward: 5´- aagctgtagagattgaggagcaaac -3´  Reverse: 5´- tcaggatgaacctggctgacta -3´ | | 60°C | 76 |
| FOXO1 | Forward: 5´- tcatgtcaacctatggcag -3´  Reverse: 5´- catggtgcttaccgtgtg -3´ | | 60°C | 131 |
| NOX4 | Forward: 5´-ggctggaggcattggagtaa -3´  Reverse: 5´-ccagtcatccaacagggtgtt -3´ | | 53°C | 61 |
| GAPDH | Forward: 5´- acagtcagccgcatcttctt -3´  Reverse: 5´- acgaccaaatccgttgactc -3´ | | 60°C | 94 |
